# Partial pulmonary embolization disrupts alveolarization in fetal sheep

**DOI:** 10.1186/1465-9921-11-42

**Published:** 2010-04-23

**Authors:** Caitlin E Filby, Stuart B Hooper, Megan J Wallace

**Affiliations:** 1 The Ritchie Centre, Monash Institute of Medical Research, Monash University, PO Box 5418, Clayton, Victoria 3168, Australia; 2 Murdoch Childrens Research Institute, Royal Children's Hospital, Parkville Victoria 3052, Australia

## Abstract

**Background:**

Although bronchopulmonary dysplasia is closely associated with an arrest of alveolar development and pulmonary capillary dysplasia, it is unknown whether these two features are causally related. To investigate the relationship between pulmonary capillaries and alveolar formation, we partially embolized the pulmonary capillary bed.

**Methods:**

Partial pulmonary embolization (PPE) was induced in chronically catheterized fetal sheep by injection of microspheres into the left pulmonary artery for 1 day (1d PPE; 115d gestational age; GA) or 5 days (5d PPE; 110-115d GA). Control fetuses received vehicle injections. Lung morphology, secondary septal crests, elastin, collagen, myofibroblast, PECAM1 and HIF1α abundance and localization were determined histologically. *VEGF-A, Flk-1, PDGF-A *and *PDGF-Rα *mRNA levels were measured using real-time PCR.

**Results:**

At 130d GA (term ~147d), in embolized regions of the lung the percentage of lung occupied by tissue was increased from 29 ± 1% in controls to 35 ± 1% in 1d PPE and 44 ± 1% in 5d PPE fetuses (p < 0.001). Secondary septal crest density was reduced from 8 ± 0% in controls to 5 ± 0% in 1d PPE and 4 ± 0% in 5d PPE fetuses (p < 0.05), indicating impaired alveolar formation. The deposition of differentiated myofibroblasts (23 ± 1% vs 28 ± 1%; p < 0.001) and elastin fibres (3 ± 0% vs 4 ± 0%; p < 0.05) were also impaired in embolized lung regions of PPE fetuses compared to controls. PPE did not alter the deposition of collagen or PECAM1. At 116d GA in 5d PPE fetuses, markers of hypoxia indicated that a small and transient hypoxic event had occurred (hypoxia in 6.7 ± 1.4% of the tissue within embolized regions of 5d PPE fetuses at 116d compared to 0.8 ± 0.2% of tissue in control regions). There was no change in the proportion of tissue labelled with HIF1α. There was no change in mRNA levels of the angiogenic factors *VEGF *and *Flk-1*, although a small increase in *PDGF-Rα *expression at 116d GA, from 1.00 ± 0.12 in control fetuses to 1.61 ± 0.18 in 5d PPE fetuses may account for impaired differentiation of alveolar myofibroblasts and alveolar development.

**Conclusions:**

PPE impairs alveolarization without adverse systemic effects and is a novel model for investigating the role of pulmonary capillaries and alveolar myofibroblasts in alveolar formation.

## Introduction

Infants born very preterm often suffer from respiratory failure at birth and require ventilatory support to survive. However, mechanical ventilation can cause lung injury and increases the risk of the infant developing bronchopulmonary dysplasia (BPD) [1]. One of the primary pathological characteristics of BPD is the presence of fewer alveoli that are larger and more simplified in structure, suggesting there has been an arrest of alveolar development [1]. To improve the therapeutic options available to these infants, it is critical to understand the factors that regulate normal and abnormal development of alveoli.

In addition to reduced alveolar development, infants with BPD also exhibit pulmonary capillary dysplasia [1] and it is possible that these two features of BPD are related. For instance, ligation of the pulmonary artery (PA) [2,3] or ductus arteriosus (DA) [4] profoundly impairs lung development, indicating that normal pulmonary blood flow is essential for normal lung development. Furthermore, inhibitors of angiogenesis and the disruption of genes involved in angiogenesis, vasculogenesis or endothelial cell maturation, also impair alveolarization [5-9]. However, those studies were complicated by either widespread systemic effects on overall fetal development, or by reduced lung liquid production which can lead to lung hypoplasia and impaired alveolar development [10]. Pulmonary hypertension is also common in very preterm infants and impairs lung growth and alveolarization when induced experimentally by prenatal ligation of the DA [4,11,12]. However, it is unclear whether pulmonary hypertension is a cause or consequence of altered pulmonary vascular development in very preterm infants and may be secondary to ventilation-induced microvascular injury.

Inactivation of the vascular endothelial growth factor-A (*VEGF-A*) gene in the respiratory epithelium of mice blocks pulmonary capillary development and causes a major defect in the formation of primary septa [13]. This demonstrates that signalling between the respiratory epithelium and pulmonary capillaries is important for primary septation. However, as these mice die within 1-2 h of birth [13], before alveolar formation commences, the relationship between alveolarization (secondary septation) and capillary development is unknown.

To study the interactions between the developing alveoli and pulmonary capillaries without inducing systemic effects, we have injected microspheres into the left pulmonary artery (LPA) of fetal sheep to disrupt the alveolar capillary bed during the alveolar stage of development. Our aim was to partially embolize the pulmonary vascular bed without causing chronic tissue hypoxia or necrosis. This study reports a new model of impaired alveolar development that will be useful in studying the interactions between the developing pulmonary vasculature and alveoli.

## Methods

### Surgical Procedure

All experiments were performed on chronically catheterized fetal sheep and were approved by the Monash University Committee for Ethics in Animal Experimentation. Aseptic surgery was performed on pregnant Merino X Border-Leicester ewes at 105-110 days gestational age (GA; term ~147d GA). Anaesthesia of the ewe and fetus was induced with thiopentane sodium (1 g) and maintained with 0.5-3% isoflurane in O_2_-N_2_O. Catheters were inserted into the fetal carotid artery, jugular vein and amniotic sac to monitor fetal well-being. Two catheters were also inserted into the fetal trachea; one directed toward the lungs and the other directed toward, but not entering the larynx. After these catheters were externalized they were connected together to form a continuous tracheal loop which allowed the normal flow of lung liquid into and out of the fetal lung [14]. An ultrasonic flow probe (4PSB, S series, Transonic Systems, USA) was placed around the left pulmonary artery (LPA) to measure pulmonary blood flow (PBF) and a catheter was inserted in the main pulmonary trunk and directed into the LPA [15]. Catheters were externalized, all incisions were closed and ewes and fetuses were allowed ~5 days recovery before commencing experiments.

### Experimental procedure

At the start of each experiment lung liquid was drained (LLD) into a sterile bag to reduce fetal pulmonary vascular resistance and increase regional PBF [16,17] which would improve the distribution of microspheres throughout the lung and minimise their loss to the systemic circulation. All fetuses received 1 ml injections into the LPA, every 10 minutes, commencing 20 minutes following LLD, of either vehicle (heparinized saline with 0.01% Tween 80) or microspheres, followed with 3 ml of heparinized saline. Each microsphere injection contained approximately 1 million, 15 μm, blue or violet microspheres (EZ Trac Ultraspheres, Interactive Medical Technologies, Stason, USA) in heparinized saline with 0.01% Tween 80. Following the injections, lung liquid was returned to the lung.

#### Study 1

Fetal sheep were randomly allocated to one of three groups. In each group, surgery was performed at ~105 or ~110 days, treatment (embolization or control) began at 110 or 115 days, and tissues were collected at 130 days of gestation.

**1. Control fetuses **underwent surgery at ~110d GA. At ~115d GA fetuses received 5-7 vehicle injections (n = 6).

**2. 1d PPE + 15d fetuses **underwent surgery at ~110d GA. At ~115d GA fetuses received 5-7 microsphere injections (n = 6).

**3. 5d PPE + 16d fetuses **underwent surgery at ~105d GA and received 5 microsphere injections per day for 5 days from ~110-115d GA (n = 5).

The PPE groups were administered 5-7 injections of microspheres on each day of the embolization period based on histological evidence demonstrating that this level of embolization did not cause necrosis. PBF was measured during embolization to measure the changes in mean PBF caused by treatment. Our aim was to transiently reduce PBF by ~25%, but in a preliminary experiment, we found that injections of up to 23 million microspheres only marginally reduced PBF.

#### Study 2

Fetal sheep were randomly allocated to one of two groups:

**1. Control fetuses, 117d GA**. Lung tissue was collected from fetuses that did not undergo surgery and from fetuses that underwent surgery at ~105d GA and received vehicle injections (n = 6 in total).

**2. 5d PPE, 116d GA fetuses **underwent surgery at ~105d GA and received 5 injections of microspheres each day for 5 days from ~110-115d GA (n = 6). At 116d GA, fetuses received an injection (i.v.) of pimonidazole hydrochloride (Hypoxyprobe-1; 85-125 mg/kg in saline; Natural Pharmacia International Inc., USA) 2 h prior to post-mortem, to determine regions of tissue hypoxia.

Fetal body weight was estimated at surgery and the actual dose of pimonidazole hydrochloride (Hypoxyprobe-1) delivered was calculated using the post-mortem body weight. Pimonidazole hydrochloride forms adducts with proteins in hypoxic cells (PaO_2 _< 10 mmHg at 37°C) [18] and these were detected later immunohistochemically.

### Post-mortem and tissue collection

At the end of *Study 1 *(at ~130d GA) and *Study 2 *(at ~116d GA), ewes and fetuses were humanely killed by an overdose of pentobarbitone sodium (6.5 g). Fetal body, lungs, heart, liver and kidney weights were recorded. The position of the LPA catheter and the location of visible microspheres were noted. In *Study 1 *the whole lung was fixed at 20 cmH_2_O via the airways with 4% paraformaldehyde. The kidneys and three cotyledons closest to the point of entry of the umbilical vessels were collected to analyse systemic microsphere distribution. Randomly collected lung tissue sections were processed for light microscopy and embedded in either paraffin or optimum cutting tissue (OCT) compound. In *Study 2 *the lungs were separated into the five lobes, weighed, cut into 1 cm^3 ^portions, frozen in liquid nitrogen and stored at -70°C.

### Histological and immunohistochemical analysis

For all histological and immunohistochemical analyses, three sections per fetus (two from the left upper and one from the left lower lobe) were cut from paraffin-embedded (5 μm), OCT-embedded (10 μm), or fresh frozen (10 μm) lung tissue. In a few fetuses, the PA catheter was inadvertently placed into the right PA and in those fetuses one section from each lobe (upper, middle, lower) of the right lung was analyzed.

#### Histological stains and immunohistochemistry

Paraffin-embedded lung tissue sections from *Study 1 *were labeled with an antibody for α-smooth muscle actin (αSMA), a marker of alveolar myofibroblasts, or stained with; haematoxylin and eosin (H&E), Hart's stain for elastin, or Gordon and Sweet's stain for reticulin which stains collagen fibres I and III, as described previously [19,20]. Paraformaldehyde-fixed OCT-embedded lung sections from *Study 1 *were labeled with an antibody for platelet endothelial cell adhesion molecule 1 (PECAM1), a marker of capillary endothelial cells; the OCT improved detection of the PECAM1 antigen. In *Study 2 *fresh frozen sections were labeled with antibodies against pimonidazole adducts (Hypoxyprobe-1) or HIF-1α (a marker of hypoxia), or stained with H&E.

Paraffin sections were deparaffinized and rehydrated. Paraffin and OCT-embedded lung tissue sections were then immersed in 0.01 M Tris/0.001 M EDTA, pH 9 and placed in a household microwave oven on high for 20 min (αSMA) or 10 min (PECAM1) to improve antigen retrieval. OCT and fresh frozen sections were air-dried at room temperature before being immersed in water. Fresh frozen sections were then post-fixed in ice-cold acetone for 10 min. All sections were then washed in PBS (3 × 5 min), endogenous peroxidases were blocked and sections were again washed in distilled water then PBS (2 × 5 min) before non-specific binding was blocked. Sections were incubated with primary antibody for 1-1.5 h at room temperature or 24 h at 4°C (PECAM1). The primary antibodies used were: monoclonal mouse anti-human αSMA (1:200; Clone 1A4, DakoCytomation, Denmark), monoclonal mouse anti-ovine PECAM1 primary antibody (1:75; Clone MCA 1097G, Serotek, UK), mouse anti-Hypoxyprobe-1 primary antibody (1:10; Natural Pharmacia International Inc., USA) and rabbit polyclonal anti-HIF-1α primary antibody (1:100; Novus Biologicals, Cat# NB100-134, USA).

Following incubation with the primary antibody, sections were washed in PBS/0.1% Tween 20 (3 × 5 min) before incubation with the secondary antibody (for HIF-1α a biotinylated goat anti-rabbit IgG, Vector Laboratories, USA was used; for all other primary antibodies a biotinylated polyclonal goat anti-mouse IgG antibody was used, DakoCytomation, Denmark). Sections were incubated with secondary antibodies (1:500 for all except HIF-1α 1:200) in DakoCytomation antibody diluent (Dako, USA) for 1 h in a humidified chamber at room temperature. Sections were again washed in PBS/0.1% Tween 20 (3 × 5 min), incubated in ABC reagent (diluted 1:150; Vectastain ABC detection, Vector Laboratories, USA) and washed in PBS (3 × 5 min). The colour reaction was developed by incubating the sections with 3,3'-Diaminobenzidine (Sigma Fast DAB peroxides substrate, Sigma, USA) for up to 7 min. Sections were washed in PBS, counterstained with haematoxylin, dehydrated, mounted and viewed under a light microscope.

#### Stereological and image analysis

Embolized lung tissue was initially visualized using a 20× objective (field of view ~1.3 mm^2^). Preliminary observations indicated that there tended to be either very few microspheres (i.e. 0-3) or a considerable number of microspheres (~10-30) within these low power fields of view, presumably reflecting regions of lung either being poorly or well-perfused, respectively, at the times of microsphere injection. Based on this observation, we chose 10 microspheres as the lower cut-off for the definition of an embolized region. Once an embolized region had been visualized, the magnification was increased and images were captured using a 100× objective. The saved images were de-identified and the following analyses were performed with the observer blinded to the group.

The percentage of lung occupied by tissue was calculated using image analysis of H&E stained lung tissue sections; for each field of view the total area of lung tissue was expressed as a proportion of total area (tissue and airspace). The density of secondary septal crests, a measure of alveolarization, was determined using sections treated with Hart's stain for elastin to identify secondary septal crests. A point counting method was used to count the number of times the point grid fell on elastin-containing crests; this was expressed as a proportion of the number of times the point grid fell on lung parenchyma for each field of view [21]. The relative abundance of elastin, collagen, αSMA, PECAM1 and Hypoxyprobe-1 adducts were determined by expressing the area of positively stained lung tissue as a proportion of parenchymal tissue area for each field of view. Positively stained lung tissue was selected by colour segmentation of the images using Image ProPlus (Media Cybernetics, USA). Differences in nuclear HIF-1α staining were determined by expressing the number of distal lung cells labelled with HIF-1α as a proportion of the total number of nuclei present within a field of view; a minimum of 3 fields of view and 1500 nuclei per animal were counted at 1000× magnification. Lung tissue from the age-matched control fetuses were used to control for all histological and immunohistochemical analyses except for the Hypoxyprobe-1 adducts. In the latter, Hypoxyprobe-1 staining was compared between non-embolized and embolized regions to account for minor differences in dose between animals caused by differences in body weight. Lung sections from a fetus made chronically hypoxic by single umbilical artery ligation (generously provided by V. Supramaniam, Monash University) were used as a positive hypoxic tissue control for the Hypoxyprobe-1 analysis.

### Gene expression levels

Total RNA was extracted from 3-5 separate pieces of lung tissue (~150 mg each) from each lobe and DNase treated using a RNeasy Midi Kit and RNase-Free DNase Set, (Qiagen, USA). To ensure that the RNA in PPE fetuses came from embolized regions of the lung, the number of microspheres per gram of tissue was determined for each RNA extraction (see below). Only RNA from regions with >30,000 microspheres/gram of lung tissue was selected for qRT-PCR. RNA (500 ng) was then reverse transcribed using random hexamers and M-MLV Reverse Transcriptase (100U; Promega, USA). *VEGF-A*, its receptor *Flk-1, platelet derived growth factor (PDGF-A) *and its receptor *PDGFR-α *and *18S rRNA *were amplified by qRT-PCR using a Mastercycler^® ^ep realplex real-time PCR system (Eppendorf, Germany) (see Table 1 for primer details). *18S rRNA *was used to adjust for minor differences in the amount of cDNA template in each reaction. Reactions containing 1 μl cDNA template (200 ng; 500 ng for *Flk-1*), 2 μl primers (10 μM), 10 μl SYBR Green (Platinum^® ^SYBR^® ^Green qPCR SuperMix-UDG; Invitrogen Life Technologies, USA) and 7 μl nuclease-free water were performed in triplicate. A control reaction containing all reagents except the cDNA template was included to ensure there was no contaminating DNA. The mean threshold (C_T_) value for each sample was calculated and subtracted from the mean C_T _value for *18S *(ΔC_T_). The ΔC_T _value was then normalized (2^-ΔCT^) and expressed relative to the mean mRNA levels of the gene of interest in the control group.

**Table 1:** Primers used for qRT-PCR

Gene of interest		Accession #, Species	Region amplified (nt#)		Primers 5'-3' F: Forward R: Reverse	Annealing **temp**. (°C)
*VEGF-A*		AF071015.1, Ovis aries	264-338		F: CGAAAGTCTGGAGTGTGTGC R: TATGTGCTGGCTTTGGTGAG	60

*Flk-1*		AF534634, Ovis aries	385-462		F: CCCAATCAGAGACCCACG R: GCCATCCTGTTGAGCGTTA	60

*PDGF-A*		NM_001075231.1, Bos taurus	784-856		F: ATGGCGTGTTACATTCCTA R: TTCACGGAGGAGAACAAAGA	59

*PDGF-Rα*		XM_590921, Bos taurus	213-276		F: GCAGGAGATCAGAGTGGAGA R: TGAAAGCTGGCAGAGGATTA	59

*18S*		X01117, Rattus norvegicus	1495-1673		F: GTCTGTGATGCCCTTAGATGTC R: AAGCTTATGACCCGCACTTAC	59-60

nt# = nucleotide numbers, temp. = temperature.

### Determination of systemic and pulmonary microsphere distribution

In *Study 1*, to determine the degree of embolization in tissues with blood flow immediately down-stream of the lung, the entire fetal kidneys and 3 cotyledons were digested and microspheres counted. Tissues (~15 g/digestion) were boiled in 15 ml of 2 M NaOH for 15 min then vortexed, repeatedly, until the solution was homogeneous. Prewarmed (70°C) washing reagent (1% ethanol/0.05% Triton X/0.1% Tween 80/0.02% sodium azide) was added to a final volume of 50 ml, mixed, then centrifuged (room temperature, 1500 rcf for 30 min) and the supernatant aspirated. In *Study 2*, pellets from RNA extractions were also washed with washing reagent. All pellets were then resuspended in 5 ml of 0.2% Tween 80/0.1% sodium dodecyl benzene sulfonate/0.02% sodium azide, vortexed, centrifuged for 15 min and the supernatant aspirated to a final volume of 1-2 ml. Ten aliquots of the extracted microsphere solution (0.9 μl each) were counted under a light microscope using a haemocytometer. For each piece of lung tissue used for RNA extraction in Study 2, this value was used to identify embolized regions of the lung (chosen as regions containing >30,000 microspheres/g lung tissue) for gene expression analysis. For each piece of kidney or cotyledon digested from Study 1, the aliquots were used to determine the total number of microspheres in each organ and to express that value as a proportion of the total number of microspheres injected. This was used to determine whether the effect of embolization was largely limited to the lungs.

### Statistical analysis

All data are expressed as mean ± standard error of the mean (SEM). Statistical significance was achieved at a p-value of <0.05. Differences in fetal body and organ weights were determined using an ANOVA while differences in stereological measurements and immunohistochemistry values were determined using a Nested ANOVA. ANOVAs were followed by the post-hoc least square difference (LSD) test. Gene expression levels were analysed by a non-paired t-test.

## Results

All fetuses were considered healthy throughout the experiments as determined from arterial blood samples (range pH 7.33-7.36, PaO_2 _18-25 mmHg, PaCO_2 _35-50 mmHg, SaO_2 _55-80%, tHb 7-11 g/dL). There were no sustained alterations in mean PBF following embolization in either study compared to the pre-embolisation period (data not shown). There were no significant differences in body weights, organ weights or lung volumes (Table 2) between control and embolized fetuses in either study, except that 1d PPE + 15d fetuses had smaller heart weights corrected for body weight compared to control fetuses. Very few of the total microspheres injected were found in the fetal kidneys (0.44 ± 0.12% and 0.43 ± 0.23%), or in the three cotyledons closest to the point of entry of the umbilical vessels (0.45 ± 0.16% and 0.90 ± 0.46%), in 1d PPE + 15d fetuses and 5d PPE + 16d fetuses, respectively.

**Table 2:** Fetal body weights (kg), organ weights (g/kg) and lung volumes (cm^3^/kg) for fetuses in PPE treatment groups compared to corresponding age-matched control group (* p < 0.05).

	*Study 1*			*Study 2*	
	**Age-matched control**, 130d GA	**1d PPE + 15d**, 130d GA	**5d PPE + 16d**, 130d GA	Age-matched **control**, 116d GA	5d PPE, 116d GA
Body wt (kg)	3.4 ± 0.1	3.2 ± 0.3	3.5 ± 0.2	2.5 ± 0.4	2.3 ± 0.2

Wet lung wt/Body wt (g/kg)	33.2 ± 1.7	33.8 ± 2.1	31.2 ± 2.8	39.4 ± 5.4	34.3 ± 3.6

Heart wt/Body wt (g/kg)	7.4 ± 0.2	6.6 ± 0.1*	6.9 ± 0.3	6.3 ± 0.3	7.6 ± 0.2*

Liver wt/Body wt (g/kg)	27.3 ± 1.4	24.5 ± 2.2	30.2 ± 2.3	27.9 ± 3.7	38.9 ± 2.3

Left Kidney wt/Body wt (g/kg)	3.1 ± 0.1	2.9 ± 0.1	3.4 ± 0.1	3.7 ± 0.2	4.0 ± 0.2

Right Kidney wt/Body wt (g/kg)	3.1 ± 0.2	2.8 ± 0.2	3.3 ± 0.1	3.8 ± 0.2	3.9 ± 0.2

Total Lung Volume/Body wt (cm^3^/kg)	27.3 ± 1.7	29.2 ± 2.9	26.8 ± 3.0	N.D.	N.D.

Left Lung Volume/Body wt (cm^3^/kg)	10.5 ± 0.7	10.4 ± 0.7	11.0 ± 1.1	N.D.	N.D.

Right Lung Volume/Body wt (cm^3^/kg)	16.7 ± 1.0	18.8 ± 2.3	15.7 ± 2.0	N.D.	N.D.

(ND = not determined)

### Morphology of the distal airways at 130d GA (Study 1)

**Figure 1:**
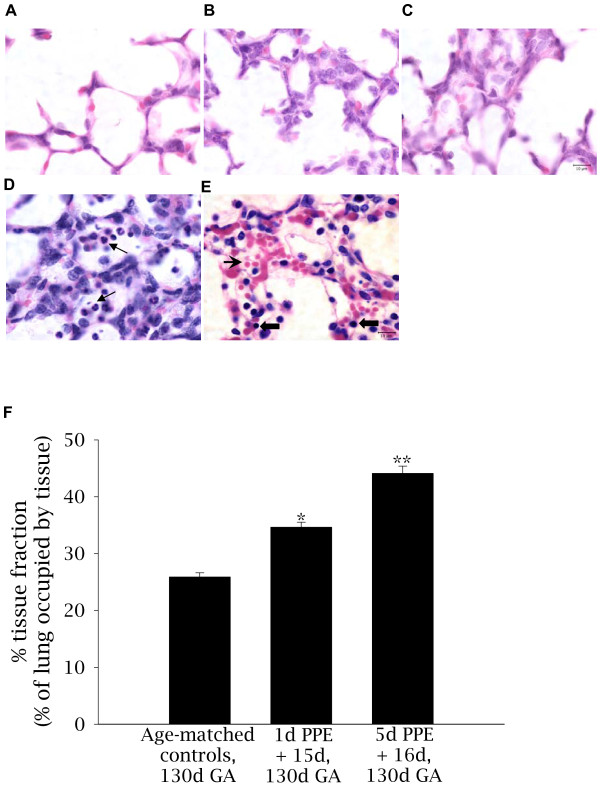
**Lung morphology and percentage tissue fraction of the lung for control and embolised fetuses**. Light micrographs stained with haematoxylin (purple: nuclei) and eosin (pink: cytoplasm) depicting lung morphology in control (A), 1d PPE + 15d (B), and 5d PPE +16d (C) lung tissue. Evidence of inflammatory cells and lung damage were only apparent in one fetus (D, E) which formed part of a pilot study and received significantly more microspheres than PPE fetuses. In that fetus, neutrophils (D, arrow), monocytes (E, thick arrows) and extravasation of erythrocytes (E, pointed arrowhead) could be seen. The scale bar in (C) represents 10 μm in all five images. The percentage tissue fraction of the lung (F) was greater in 1d PPE + 15d than in control fetuses (* p < 0.001) and greater in 5d PPE + 16d fetuses than in 1d PPE + 15d or control fetuses (** p < 0.001).

Following embolization, the distal airways had thicker lung parenchyma and fewer, simplified air sacs in comparison to age-matched controls (Figure 1A-C). However, the areas of lung tissue affected by embolization were not uniform throughout the entire lung. Embolized areas, determined by the presence of ~10-30 microspheres in low power fields of view, occurred in discrete regions and occupied in total ~20% of the lung in 1d PPE + 15d fetuses and ~30% of the lung in the 5d PPE + 15d fetuses. These embolized regions had altered morphology, while the intervening, non-embolized areas appeared unaffected. In PPE fetuses therefore, although sections were selected randomly, only embolized regions of the lung were analysed and compared to lung tissue from control fetuses, while non-embolized regions (containing <10 microspheres) were excluded from the analysis.

In control and embolized fetal lung tissue, no histological signs of inflammation or necrosis were observed. In contrast, a fetus that received 23 million microspheres (over a 5 h period) as part of a pilot study, had evidence of marked septal thickening and lung damage. This included extravasation of erythrocytes and infiltration of inflammatory cells, particularly neutrophils and monocytes (Figure 1D-E).

#### Percentage of lung occupied by tissue

The percentage of lung occupied by tissue at 130d GA in embolized regions of lung, was significantly increased from 28.6 ± 0.7% in control fetuses to 34.6 ± 0.9% in 1d PPE + 15d fetuses (p < 0.001) and further increased to 44.1 ± 1.3% in 5d PPE + 16d fetuses (p < 0.001, Figure 1F).

#### Localization and relative abundance of elastin

**Figure 2:**
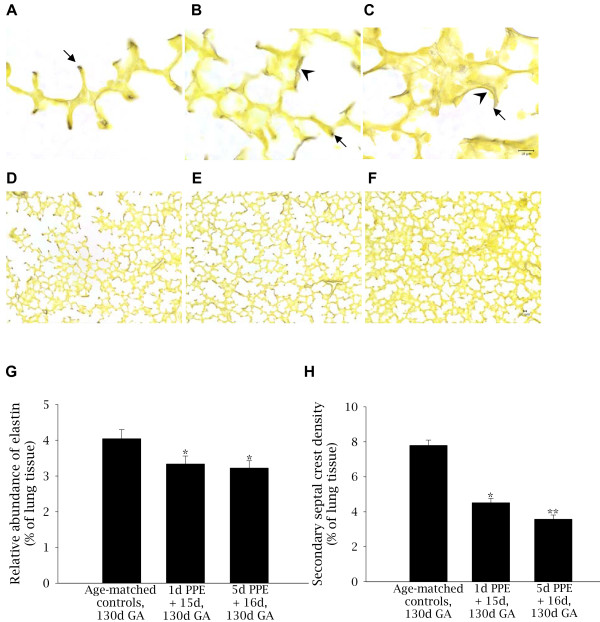
**Localization and relative abundance of elastin and density of secondary septal crests in control and embolised fetal lung tissue**. Light micrographs depicting the localization of elastin (purple-black) staining in control (A), 1d PPE + 15d (B), and 5d PPE + 16d (C) lung tissue. Nuclei and cytoplasm are counterstained yellow. The arrows in A-C indicate secondary septal crests and the arrow-heads indicate deposition of elastin throughout the primary septal wall and around the base of alveoli in PPE fetuses (B-C). The scale bar in (C) represents 10 μm in images A-C and the scale bar in F represents 10 μm in images D-F. The relative abundance of elastin (G) was decreased in 1d PPE + 15d and 5d PPE + 16d fetuses relative to control fetuses (* p < 0.05). The density of secondary septal crests in the lung parenchyma (H) of 1d PPE + 15d fetuses was lower than in control fetuses (*p < 0.001) and lower in 5d PPE + 16d fetuses relative to 1d PPE + 15d fetuses and control fetuses (**p < 0.05).

In control fetuses, elastin staining in the peri-alveolar lung parenchyma was primarily located in discrete bundles at the tips of secondary septal crests, although thin ribbon-like fibres were observed in some alveolar walls (Figure 2A). Elastic fibres were not commonly located at the base of the alveoli in control fetuses. In contrast, embolized regions of the lung in PPE fetuses had stunted secondary crests and elastic fibres were commonly deposited throughout the primary septal wall and at the base of alveoli (Figure 2B-C). The proportion of distal lung tissue stained for elastin (relative abundance of elastin) was significantly less in embolized regions of fetuses exposed to 1d PPE + 15d (3.3 ± 0.2%) and 5d PPE + 16d (3.2 ± 0.2%) compared with control fetuses (4.0 ± 0.3%, p < 0.05, Figure 2G).

#### Secondary septal crest density

Light micrographs, stained for elastin were used to locate secondary septal crests in control and embolized fetuses. At 130d GA, the secondary septal crests in control fetuses were in various stages of formation. Most were elongated, mature secondary septal crests with large bundles of elastin fibres present at the tips of the septa (Figures 2A &2D). In 1d PPE + 15d and 5d PPE + 16d fetuses, the morphology of secondary septal crests ranged from normal mature septal crests, to stunted in length or abnormally shaped (Figures 2B-C &2E-F). Septal crest density decreased from 7.8 ± 0.3% in control fetuses to 4.5 ± 0.2% in embolized regions of 1d PPE + 15d fetuses (p < 0.001) and to 3.6 ± 0.2% in 5d PPE + 16d fetuses (p < 0.05 relative to control and 1d PPE + 15d fetuses, Figure 2H).

#### Localization and relative abundance of collagen

Collagen staining was similar in the peri-alveolar parenchyma of control fetuses and embolized regions of PPE fetuses; it was located within primary and secondary septal walls and at the tips of secondary septal crests (data not shown). The proportion of distal lung tissue stained for collagen fibres (relative abundance of collagen) was similar in all groups: 16.9 ± 0.8% in control fetuses, 18.4 ± 0.9% in 1d PPE + 15d fetuses and 15.8 ± 0.8% in 5d PPE + 16d fetuses.

#### Alveolar myofibroblasts - localization and relative abundance of αSMA

**Figure 3:**
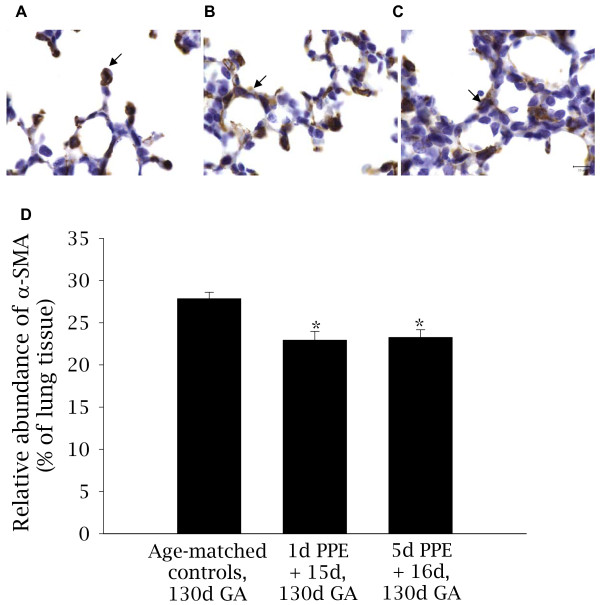
**Localization and relative abundance of αSMA in control and embolized fetal lung tissue**. Light micrographs depicting the localization of αSMA (brown-black staining); a marker of myofibroblasts, in control (A), 1d PPE + 15d (B), and 5d PPE + 16d (C) lung tissue. Nuclei are counterstained blue with haematoxylin. The arrows show the presence of αSMA at the tips of secondary septal crests (A) and in the primary septal wall (B-C). The scale bar in (C) represents 10 μm in all three images. The relative abundance of αSMA (D) was less in 1d PPE + 15d and 5d PPE + 16d fetuses relative to control fetuses (* p < 0.001).

Alveolar myofibroblasts in the peri-alveolar region of the lung were detected using an antibody against αSMA. In control fetuses, αSMA in the distal lung parenchyma was primarily localized to secondary septal crests, although some myofibroblasts were adjacent to the primary septal wall (Figure 3A). In contrast, in embolized fetuses (Figure 3B-C), αSMA was located in stunted secondary septal crests and to a greater degree in the primary septal wall. The relative abundance of αSMA within the lung parenchyma was significantly lower in embolized regions of the lung in 1d PPE + 15d fetuses (22.9 ± 1.0%) and 5d PPE + 16d fetuses (23.3 ± 0.9%) relative to control fetuses (27.8 ± 0.7%, p < 0.001, Figure 3D).

#### Pulmonary capillary development - localization and relative abundance of PECAM1

**Figure 4:**
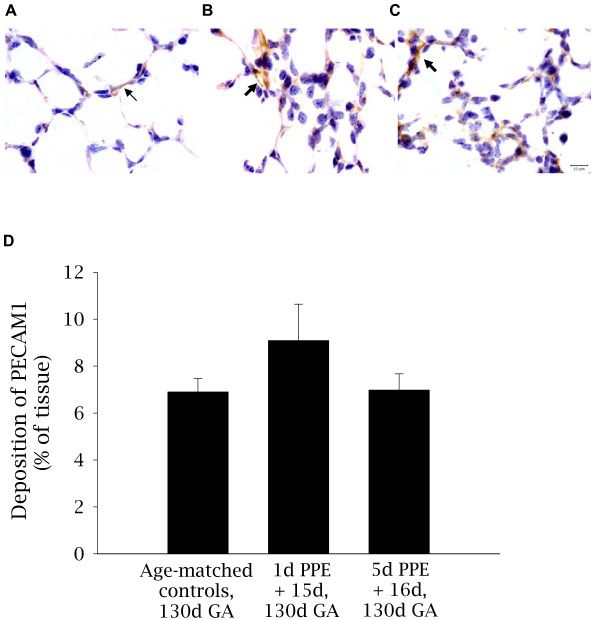
**Localization and relative abundance of PECAM1 in control and embolized fetal lung tissue**. Light micrographs depicting the localization of PECAM1 (brown staining); a marker of endothelial cells, in control (A), 1d PPE + 15d (B), and 5d PPE +16d (C) lung tissue. Nuclei are counterstained blue with haematoxylin. The thin arrow indicates normal pulmonary capillaries (A) and the thick arrows indicate abnormal capillary staining in the parenchyma of 1d PPE + 15d and 5d PPE +16d fetuses (B-C). The scale bar in (C) represents 10 μm in all three images. The relative abundance of PECAM1 (D) was not significantly different between groups. However, the PPE fetuses appeared to have larger capillaries compared to control fetuses and PECAM1 staining was less common within the septal walls of PPE fetuses.

In control fetuses, light PECAM1 staining identified the small capillaries in both the primary and secondary septal walls (Figure 4A). In contrast, embolized regions of lung from 1d PPE + 15d fetuses (Figure 4B) PECAM1 staining was less common within the secondary septal walls. Embolized regions of lung from 5d PPE + 16d fetuses showed PECAM1 in the thickened primary septal walls (Figure 4C). The relative abundance of PECAM1 in the distal lung parenchyma was 6.9 ± 0.6% in control fetuses which was similar to embolized regions of 1d PPE + 15d (9.1 ± 1.6%) and 5d PPE + 16d (7.0 ± 0.7%) fetuses (Figure 4D).

### Markers of hypoxia and vascular development at 116d GA (Study 2)

#### Changes in regional lung tissue hypoxia

The proportion of lung cells positively stained for HIF1α was not different in embolized areas of lung in 5d PPE fetuses at 116d GA (29.0 ± 11.4%) in comparison to control fetuses (27.9 ± 11.8%). There was also no evidence of inflammatory cells in H&E stained lung tissue sections from 5d PPE fetuses at 116d GA or in age-matched controls.

**Figure 5:**
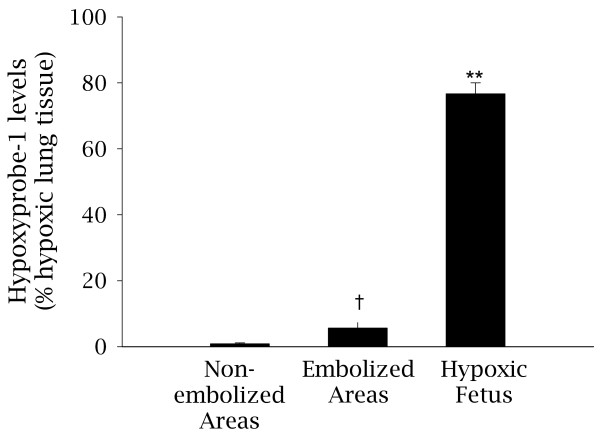
**Levels of Hypoxyprobe-1 pimonidazole adducts in lung tissue from 5d PPE fetuses**. The levels of hypoxyprobe-1 pimonidazole adducts, a marker of hypoxic tissue (PaO_2 _<10 mmHG) was determined in embolized and non-embolized areas of lung tissue from 5d PPE fetuses († p < 0.05 relative to embolized and non-embolized regions of 5d PPE fetuses). As a comparison, the level of Hypoxyprobe-1 in a severely hypoxic fetus exposed to single umbilical artery ligation is shown (**p < 0.001).

Pimonidazole adducts were used as a sensitive method of assessing whether the embolized regions were hypoxic. Two fetuses were larger than expected at post-mortem so the dose of pimonidazole hydrochloride administered (41 mg/kg and 73 mg/kg) was not sufficient for adduct detection. The remaining four fetuses received 106 ± 8.1 mg/kg, which was sufficient for adduct detection. The proportion of distal lung tissue stained for Hypoxyprobe-1 pimonidazole adducts (% hypoxic lung tissue) was very low in control regions (0.8 ± 0.2%; Figure 5). Although Hypoxyprobe-1 staining was significantly increased (p < 0.05) in embolized regions of the lung compared to control areas, only 6.7 ± 1.4% of embolized lung tissue had detectable levels of hypoxia. In comparison, in a fetus made chronically hypoxic (arterial PO_2 _of 14.3 mmHg) due to single umbilical artery ligation, the percentage of hypoxic lung tissue was 76.7 ± 4.7%.

#### mRNA levels of genes that regulate vascular and myofibroblast development

**Figure 6:**
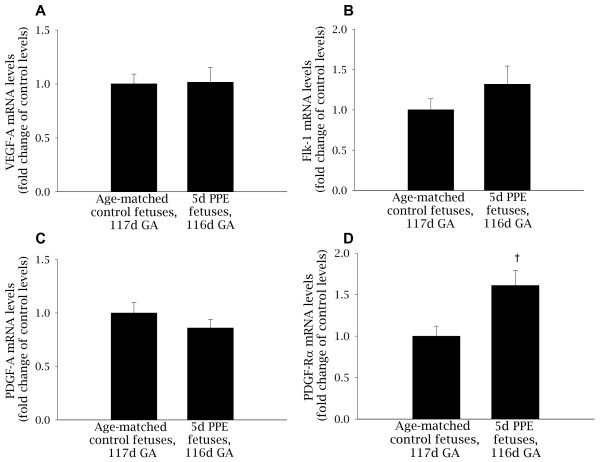
**The mRNA levels of *VEGF-A*, *Flk-1*, *PDGF-A *and *PDGF-Rα* in control and 5d PPE fetuses**. The mRNA levels of *VEGF-A *(A), *Flk-1 *(B), *PDGF-A *(C) and *PDGF-Rα *(D) in 5d PPE and control fetuses corrected for the levels of the house-keeping gene *18S *and expressed as fold change from the mean value in age-matched control fetuses. † p < 0.05.

The mRNA levels of *VEGF-A *(1.02 ± 0.14 vs 1.00 ± 0.09), the VEGF receptor *Flk-1 *(1.32 ± 0.22 vs 1.00 ± 0.14) and *PDGF-A *(0.86 ± 0.08 vs 1.00 ± 0.10) were similar at 116dGA in embolized lung regions of 5d PPE and control fetuses, respectively (Figure 6). In contrast, the mRNA levels of the PDGF receptor *PDGF-Rα *were significantly increased in embolized regions of the lung in 5d PPE fetuses (1.61 ± 0.18) compared to control fetuses (1.00 ± 0.12; p < 0.05; Figure 6) at 116d GA.

## Discussion

The results of this study indicate that perturbations to pulmonary capillary blood flow, induced by PPE impair alveolar formation during the alveolar stage of lung development. The effect of PPE on alveolar formation occurs in the absence of significant embolization in other vascular beds and occurs without causing necrosis, significant chronic lung tissue hypoxia or inflammation. The impairment of alveolarization is, therefore, likely to result from disrupted mesenchymal-epithelial signalling. The observed increase in *PDGF-Rα *mRNA levels may play a role in altered mesenchymal-epithelial signalling and warrants further investigation. PPE is, therefore, a novel experimental model that may allow elucidation of the endothelial-epithelial interactions that regulate alveolar development.

To study the interaction between developing capillaries and alveoli, previous studies have used inhibitors of angiogenesis [5,22,23] or transgenic alterations in angiogenic mediators [6,24,25]. However, these treatments caused significant systemic effects on multiple organ systems, thereby complicating the interpretation of the data. Similarly, other models are complicated by one or more of the following factors that in themselves could alter alveolar development; reductions in fetal oxygenation status, cessation of lung liquid production and impaired lung growth (PA ligation) [2,3], pulmonary hypertension (DA ligation) [4], or removal of the physicochemical environment of the lung that is essential for normal lung growth (lung allographs [26] and explants in culture [27]). In contrast, the PPE model does not alter fetal oxygenation, fetal growth or fetal lung growth and because the ductus arteriosus remains open, PPE cannot induce pulmonary hypertension. PPE therefore provides a model of impaired alveolarization that is not confounded by other changes in overall fetal or lung growth. With regard to our results, it is of interest that a pulmonary epithelial cell-specific *VEGF-A *null mouse has a major defect in the formation of primary septa which becomes lethal after birth [13]. However, as alveolar formation does not normally commence until days after birth in mice, the relationship between alveolarization (secondary septation) and capillary development could not be tested in those mice.

### Development of the PPE model

PPE is a novel model of pulmonary embolization in fetal sheep. We and others commonly use microspheres to assess instantaneous blood flow to organs like the fetal lung [16] and to embolize organs like the placenta [28], however, to our knowledge, this is the first model of fetal lung embolization *in vivo*. To specifically target the pulmonary capillary bed, we used small diameter microspheres (15 μm) to block capillaries, but not arterioles; in blocking the capillaries we did not affect mean pulmonary blood flow or lung weights. A small reduction in fetal heart weight was detected in the 1d PPE +15d group. However, as there were very few microspheres in the vascular beds immediately downstream of the lung, the small reduction in heart weight is unlikely to be related to embolization. The long gestation length of fetal sheep also gave us the opportunity to examine the effect of embolization up to 2 weeks after treatment, allowing sufficient time for the effect on alveolarization to fully manifest. No evidence of necrosis or inflammation was observed with embolization, except in one fetus that received 23 million microspheres (over 5 h) during a pilot study. Hence, capillary embolization impairs alveolarization without inducing tissue death, necrosis or overt inflammation. The main limitation of the PPE model is that the embolization is regional, which is likely due to cyclical changes in regional pulmonary perfusion [29], necessitating the identification of embolized regions.

### PPE and alveolar development

PPE appears to significantly delay lung maturation as indicated by an increase in lung parenchymal thickness, reduced secondary septal crest formation as well as a reduced and altered spatial pattern of elastin deposition. This demonstrates that alveolarization was significantly impaired by PPE and that the degree of impairment was greater with increased duration of embolization. The spatial pattern of elastin deposition was also found to be altered, with more elastin fibres located around the primary septal walls following PPE. The percentage of lung tissue stained for elastin was reduced in embolized areas, however, this may have been due to an increase in parenchymal tissue volume rather than to a reduction in the amount of elastin *per se*. Regardless, the alteration in the site of elastin deposition, combined with an increase in tissue and a reduction in the relative amount of elastin per tissue area indicates that the biomechanical properties of the lung may also be impaired following PPE. A similar pattern of elastin deposition occurs in the lungs of preterm sheep following ventilation-induced lung injury [19,21].

As alveolar myofibroblasts deposit elastin and other ECM components within the secondary septa they play an integral role in the development of the distal gas exchange structures, particularly alveoli [30]. Alpha smooth muscle actin (αSMA) is commonly used as a marker of alveolar myofibroblasts and was reduced in 1d PPE + 15d and 5d PPE + 16d fetuses. This suggests that PPE reduced differentiation of peri-alveolar fibroblasts into myofibroblasts. In addition, although alveolar myofibroblasts were generally found within the secondary septa of control lung tissue, they were found dispersed within the primary septal wall following PPE. We suggest that PPE impaired alveolar myofibroblast differentiation at the site of secondary septal crest formation, contributing to the altered spatial pattern of elastin deposition and stunted growth of secondary septal crests. These results are consistent with the suggestion that alveolar myofibroblasts are integral to signalling between the capillary endothelium and the developing secondary septal crests [30].

### PPE and pulmonary vascular development

In embolized regions of the distal lung parenchyma, the relative abundance of PECAM1 staining was not altered, however, there appeared to be fewer capillaries located within secondary septa. It is possible that that PPE induced compensatory pulmonary capillary development to sustain oxygen and nutrient delivery, but disrupted the normal developmental pattern of alveolar capillary formation. Further studies may elucidate this proposed capillary remodelling using scanned vascular casts. In a previous study, complete ligation of the LPA induced compensatory vascular growth in the lung from the systemic circulation [31]; this must have occurred very rapidly to prevent complete necrosis of the left lung and demonstrates the lung's rapid capacity for the formation of a collateral blood supply. In our less severe PPE model, it is possible that a collateral blood supply developed from adjacent non-embolized small vessels rather than from the systemic circulation. Indeed, microvascular endothelial cells isolated from the lungs of young rats (10-15 weeks old) have a much greater proliferative and vasculogenic potential than endothelial cells derived from the pulmonary artery of the same animal [32]. Hence, the capacity for vascular remodelling and growth is likely to be much greater in the microvasculature, where embolization occurred, than in the larger vessels. The altered alveolar development in the current study is consistent with lung pathologies seen in humans with alveolar capillary dysplasia [33], persistent pulmonary hypertension of the newborn [4,33] and disrupted alveolarization in infants with BPD [34]. Infants with BPD have a reduction in parenchymal capillaries and those present are often enlarged and located distant to the air-tissue interface [35].

### Mechanisms by which PPE may impair alveolar development

The proportion of hypoxic tissue detected by Hypoxyprobe-1 was increased in PPE fetuses, however this represented <7% of the embolized lung tissue. In comparison, ~77% of lung tissue bound Hypoxyprobe-1 in the chronically hypoxemic fetus (PaO_2 _of 9-16 mmHg) [36], suggesting that PPE induces very little tissue hypoxia. The lack of an increase in nuclear HIF-1α and the absence of inflammatory cell infiltration and necrosis indicates that there was no or only a minor transient biological response to the tissue hypoxia detected in embolized regions. Nevertheless, it may be sufficient to provide a stimulus for revascularisation, which is a likely consequence of PPE, as there was no overall reduction in capillary density within the alveolar region at 130d GA. It is possible that re-vascularisation was mediated by a transient increase in HIF-1α levels before tissue collection, enabling activation of HIF-1α target genes such as *VEGF-A*.

There is substantial evidence in support of a role for VEGF-A and its receptor Flk-1 in mediating critical interactions between the capillary endothelium and alveolar epithelium [13,24,37-39]. As PPE physically disrupts association of the capillaries (the site of *Flk-1 *expression) with the epithelium (the site of *VEGF-A *expression) [37,40], we considered it likely that the VEGF:Flk-1 pathway would be affected by PPE. The lack of change in *VEGF/Flk-1 *expression was therefore surprising. However, it is possible that changes in *VEGF *mRNA levels in the developing septa were masked by the high basal levels of *VEGF *expression in vascular smooth muscle cells [41], or that changes occurred prior to tissue collection, or that VEGF/Flk-1 protein levels were elevated or released from the ECM leading to receptor activation without detectable changes in gene expression.

*PDGF-Rα *mRNA levels were significantly increased by 5d PPE indicating that PDGF-A:PDGF-Rα signaling may be altered. During saccular and alveolar development, *PDGF-A *is predominately expressed by the lung epithelium and *PDGF-Rα *is expressed by alveolar myofibroblasts which deposit elastin at the tips of secondary septal crests [30,42]. In *PDGF-A *null mice, myofibroblasts fail to differentiate, alveolar elastin deposition is inhibited and secondary septation is impaired [30,42]. Surprisingly, over-expression of *PDGF-A *in mouse lung epithelium also leads to abnormal elastin deposition and to impaired saccule development [43]. This suggests that PDGF-A and its receptor may be a paracrine ligand:receptor pair involved in myofibroblast differentiation, elastin deposition and alveolarization and that their expression levels must be tightly regulated to enable correct development of the lung's gas exchange regions. The mechanism by which this occurs is not known. However, PDGF-B and PDGF-Rβ cause recruitment of pericytes (smooth muscle cells) to developing blood vessels [44,45] and mesangial cells to glomeruli [46,47]. Hence it is possible that in the lung, epithelial derived PDGF-A may recruit PDGF-Rα positive alveolar myofibroblasts to the site of secondary septation. Clearly, these results indicate that further investigation of PDGF-A:PDGF-Rα protein levels and its downstream signaling pathway within alveolar myofibroblasts during alveolarization is warranted.

## Conclusions

The results of this study demonstrate that PPE disrupts alveolarization. PPE specifically targets disruption of the pulmonary capillary bed and impairs alveolarization without reducing PBF or inducing sustained hypoxia, inflammation or necrosis. It is also possible that PPE disrupted signalling between the capillary endothelium, the alveolar myofibroblasts and the pulmonary epithelium via changes in the PDGF-A/PDGF-Rα pathway. Future application of the PPE model will be useful in investigating the interactions between the developing capillaries and the alveoli. Such studies may increase our understanding of the pathogenesis of BPD, and improve the therapeutic options available for infants with BPD.

## Competing interests

The authors declare that they have no competing interests.

## Authors' contributions

All authors devised the study concept. MW devised the model. CF developed the model, carried out the experiments and analyzed the data. MW and CF wrote the manuscript. SH and MW supervised the study. All authors read, made intellectual contributions to, edited and approved the manuscript.
